# Clinical benefit analysis of PD-1 inhibitors in patients with advanced, recurrent or metastatic cervical cancer: a meta-analysis and systematic review

**DOI:** 10.3389/fimmu.2024.1305810

**Published:** 2024-01-24

**Authors:** Yun-zi Wang, Ji-sheng Wang, Jiang Du, Xue-li Tang, Jing-ping Xiao

**Affiliations:** ^1^ Department of Pathology, Sichuan Science City Hospital, Mianyang, Sichuan, China; ^2^ Department of Pharmacy, The Third Hospital of Mianyang, Sichuan Mental Health Center, Mianyang, Sichuan, China; ^3^ Department of General Surgery, Sichuan Science City Hospital, Sichuan, China; ^4^ Department of Science and Technology, The Third Hospital of Mianyang, Sichuan Mental Health Center, Mianyang, Sichuan, China

**Keywords:** cervical cancer, programmed cell death protein-1, objective response rate, adverse events, combination

## Abstract

**Purpose:**

This study aims to comprehensively evaluate the efficacy and safety of programmed cell death protein-1 (PD-1) in patients with advanced, recurrent, or metastatic cervical cancer (ARMCC) and identify the population that may benefit the most.

**Methods:**

We conducted a search of PubMed, EMBASE, and the Cochrane Collaboration Library from their inception to September 2023. We extracted and analyzed the results related to the efficacy and safety of PD-1 in patients with ARMCC. The primary endpoints included the overall objective response rate (ORR) and adverse events (AEs), while the secondary endpoints encompassed the 1-year overall survival (OS) rate, 1-year progression-free survival (PFS) rate, as well as OS and PFS. We used a random effects model to conduct a meta-analysis on single-group rates, and the Mantel-Haenszel method was utilized to compare the ORR and the incidence of AEs.

**Results:**

Our study included a total of 21 trials involving 2,097 patients. The ORR of the combination of PD-1 inhibitors with chemotherapy was 56.36%, the combination of PD-1 inhibitors with anti-angiogenic agents was 38.72%, the combination of PD-1 inhibitors with Cytotoxic T-lymphocyte antigen 4 inhibitors was 25.60%, and PD-1 inhibitor monotherapy was 15.99%. The subgroup analysis showed that the group of patients with squamous cell carcinoma (SCC) exhibited a significantly higher ORR compared to the non-SCC group in patients who received PD-1 inhibitors combined with other anti-tumor drugs (Odds Ratio =2.43, P=0.002). Additionally, the group of patients with a programmed death-ligand 1 combined positive score (PD-L1 CPS) ≥1 exhibited a significantly higher ORR compared to the PD-L1 CPS <1 group in patients who received PD-1 inhibitor monotherapy (OR=4.14, P=0.02). PD-1 inhibitor monotherapy or PD-1 inhibitors combined with chemotherapy did not significantly increase the incidence of all grades of adverse events (Relative Risk=0.99, p=0.788) or the incidence of serious adverse events (RR=0.99, p=0.788) compared to chemotherapy alone.

**Conclusion:**

PD-1 inhibitors demonstrate outstanding efficacy in the treatment of patients with ARMCC. Patients with SCC may benefit more from treatments including PD-1 inhibitors in combination with other anti-tumor drugs, and PD-L1 CPS ≥1 can be considered a favorable indicator of immune therapy response. Importantly, the use of PD-1 inhibitor monotherapy or PD-1 inhibitors in combination with chemotherapy did not lead to an increased incidence of AEs compared with chemotherapy alone, indicting safety during treatment.

**Systematic Review Registration:**

PROSPERO (CRD42023457945).

## 1 Introduction

Cervical cancer (CC) is one of the most common malignancies among women and ranks fourth among all cancer-related deaths worldwide (1). In 2020, there were over 600,000 newly diagnosed cases of CC, with approximately 342,000 deaths, and the number of women under the age of 65 years being diagnosed with CC is also steadily increasing (2). In clinical settings, the treatment of patients with advanced, recurrent, or metastatic cervical cancer (ARMCC) is even more challenging. Despite various treatment options currently available for CC, including surgery, radiation therapy, chemotherapy, targeted therapy, and combination therapies (3), these approaches have limited survival rates and treatment effectiveness in patients with ARMCC. Therefore, there is an urgent need to develop novel therapeutic strategies.

Programmed cell death protein-1 (PD-1) inhibitors, which are a type of immunotherapy, have made significant breakthroughs in cancer treatment in recent years (4). PD-1 inhibitors inhibit the interaction between tumors and immune cells, enabling the patient’s immune system to better recognize and attack cancer cells. This novel class of drugs has been widely used to treat various types of cancers, including melanoma, lung cancer, and renal cell carcinoma, and has demonstrated remarkable clinical efficacy (5–7).

Some clinical studies have demonstrated the significant efficacy and survival advantages of PD-1 inhibitors in the treatment of cervical cancer, and current research is increasingly focusing on whether the combination of PD-1 inhibitors with other anti-tumor drugs can have better therapeutic effects in the treatment of CC.

However, no studies have investigated which specific anti-tumor drug, when combined with PD-1 inhibitors, yields the most effective results in the treatment of cervical cancer. This study aimed to provide comprehensive evidence of the efficacy of PD-1 inhibitors combined with other anti-tumor drugs in treating patients with ARMCC and to identify the patient population that benefits the most. Additionally, a comprehensive analysis of all adverse events (AEs) mentioned in the included studies was performed. This approach can provide clinicians with more accurate data and guidance when making decisions relating to treatment, ultimately leading to improved treatment strategies.

## 2 Materials and methods

### 2.1 Data sources and search strategy

This study was rigorously evaluated using the Preferred Reporting Items for Systematic Reviews and Meta-Analyses (PRISMA) guidelines (8). PubMed, EMBASE, and the Cochrane Collaboration Library databases were searched from their inception to September 2023, and the language was restricted to English. An additional search of the gray literature was performed using Google Scholar, OpenGrey, ClinicalTrials.gov, and The Cochrane Central Register of Controlled Trials.

We adjusted the medical subject headings terms combined with the related text words to comply with the rules for searching for relevant studies in each database. Our search strategy was as follows: (Cervical OR Cervix OR Cervical Neoplasm, Uterine OR Neoplasm, Uterine Cervical OR Uterine Cervical Neoplasm OR Neoplasms, Cervical OR Cervical Neoplasms OR Cervical Neoplasm OR Neoplasms, Cervix OR Cervix Neoplasm OR Neoplasm, Cervix OR Cervix Neoplasms OR Cancer of the Uterine Cervix OR Cancer of the Cervix OR Cervical Cancer OR Cancer, Cervical OR Cervical Cancers OR Uterine OR Cervical Cancer OR Cancer, Uterine Cervical OR Cervical Cancer, Uterine OR Uterine Cervical Cancers OR Cancer of Cervix OR Cervix Cancer OR Cancer, Cervix) and (PD-1 OR PD-1 inhibitors OR Programmed cell death protein-1 inhibitor). For example, the search query in PubMed was (PD-1[Title/Abstract] OR PD-1 inhibitors[Title/Abstract] OR Programmed cell death protein-1 inhibitors[Title/Abstract] OR PD-L1[Title/Abstract] OR PD-L1 inhibitors[Title/Abstract] OR Programmed Death-Ligand 1 inhibitors[Title/Abstract]) AND (Cervical[Title/Abstract] OR Cervix[Title/Abstract] OR Cervical Neoplasm, Uterine[Title/Abstract] OR Neoplasm, Uterine Cervical[Title/Abstract] OR Uterine Cervical Neoplasm[Title/Abstract] OR Neoplasms, Cervical[Title/Abstract] OR Cervical Neoplasms[Title/Abstract] OR Cervical Neoplasm[Title/Abstract] OR Neoplasms, Cervix[Title/Abstract] OR Cervix Neoplasm[Title/Abstract] OR Neoplasm, Cervix[Title/Abstract] OR Cervix Neoplasms[Title/Abstract] OR Cancer of the Uterine Cervix[Title/Abstract] OR Cancer of the Cervix[Title/Abstract] OR Cervical Cancer[Title/Abstract] OR Cancer, Cervical[Title/Abstract] OR Cervical Cancers[Title/Abstract] OR Uterine[Title/Abstract] OR Cervical Cancer[Title/Abstract] OR Cancer, Uterine Cervical[Title/Abstract] OR Cervical Cancer, Uterine[Title/Abstract] OR Uterine Cervical Cancers[Title/Abstract] OR Cancer of Cervix[Title/Abstract] OR Cervix Cancer[Title/Abstract] OR Cancer, Cervix[Title/Abstract]).

### 2.2 Study selection

Two independent researchers (Jing-ping Xiao and Yun-zi Wang) filtered the titles and abstracts of all of the retrieved studies to identify potentially relevant studies. The full texts of the retrieved studies that met the inclusion criteria were evaluated. Each of these discrepancies was resolved through discussion, and if conflicts remained, a third reviewer (Ji-sheng Wang) was consulted.

### 2.3 Inclusion and exclusion criteria

The inclusion criteria for the studies in the systematic review on the efficacy and safety of PD-1 inhibitors for the treatment of patients are as follows: (1) interventions included the use of PD-1 inhibitors; (2) Patients were ≥18 years of age; and (3) patients had a histological diagnosis of ARMCC. (4) The following outcomes were reported: objective response rate (ORR), 1-year overall survival (OS) rate, 1-year progression-free survival (PFS) rate, hazard ratios (HRs) of OS or PFS, and AEs. Editorials, meeting reports, and letters to the editors were excluded from the review. The focus was solely on primary research studies that reported specific outcomes and AEs to ensure the reliability and relevance of the findings.

### 2.4 Data extraction

Two researchers, Jing-ping Xiao and Yun-zi Wang, independently screened the studies using the predefined inclusion criteria. Any discrepancies were resolved through a consensus between the two researchers. From each included study, relevant information, such as study characteristics, baseline characteristics, and predefined outcomes, including ORR, 1-year OS rate, 1-year PFS rate, HRs for OS or PFS, and AEs (if applicable), were directly extracted from the original report.

### 2.5 Quality assessment

Two researchers, Jing-ping Xiao and Yun-zi Wang, independently used the Cochrane Risk of Bias Tool to assess the quality of eligible randomized controlled trials (RCTs) (9). The researchers also utilized the Institute of Health Economics Quality Appraisal (IHE QA) checklist (10) to evaluate the quality of eligible observational studies, which included 20 items. If a study met 14 or more items on the Delphi checklist, it was considered acceptable. Additionally, the Newcastle-Ottawa Scale (NOS) (11) was used to evaluate the quality of eligible expansion cohort studies by assessing selection, comparability, and exposure. The NOS scale included nine points, and a score of 7 or higher was considered indicative of high quality, while a score of 4–6 indicated good quality, and a score of 3 or less indicated low quality. Any discrepancies were resolved through discussion involving a third reviewer (Ji-sheng Wang) if conflicts remained.

### 2.6 Data synthesis and analysis

The primary endpoints included ORR and AEs, while the secondary endpoints included 1-year OS rate, 1-year PFS rate, OS, and PFS. The random-effects model was used to conduct a meta-analysis of single-group rates, including ORR, 1-year OS rate, and 1-year PFS rate. The Mantel-Haenszel method was used to compare the ORR stratified by programmed death-ligand 1 combined positive score (PD-L1 CPS) or histological types of squamous cell carcinoma (SCC) as well as the incidence of AEs in RCTs. The results were reported as odds ratio (OR) and relative risk (RR) with a corresponding 95% confidence interval (CI). If the I^2^ value was greater than or equal to 50%, a random-effects model was used to merge the results; otherwise, the fixed-effects model was used. I^2^ statistics were used to assess heterogeneity across the included trials, and I^2^ values of 25%, 50%, and 75% indicated low, moderate, and high inconsistencies, respectively. The continuity correction method was applied by adding a correction of 0.5 to cells with zero values. Stata (version 14) software was used to analyze all results, and statistical significance was defined as a two-sided p-value of <0.05.

## 3 Results

### 3.1 Literature search

**Figure 1:**
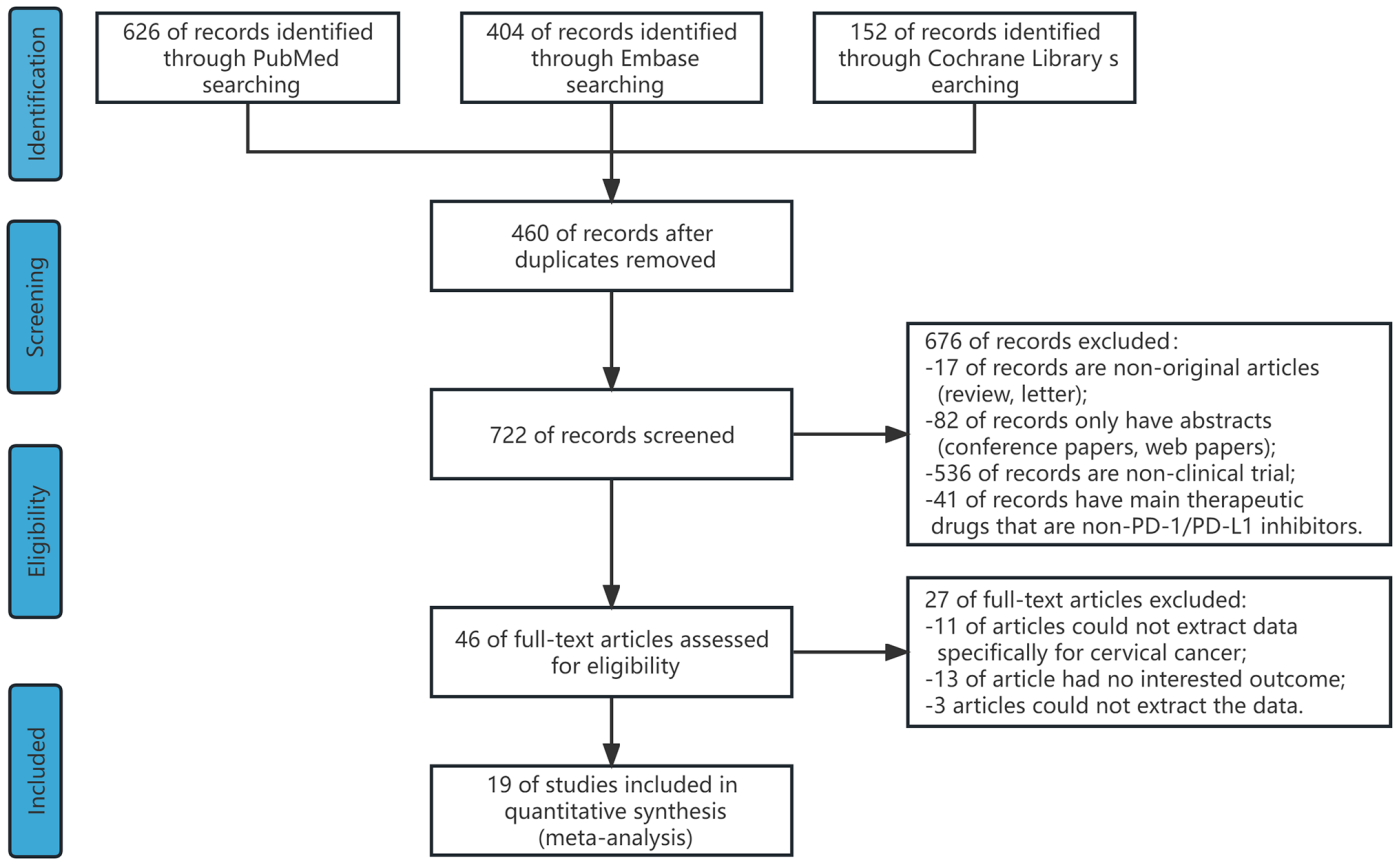
The flow diagram of studies included in this meta-analysis.

**Figure 1** displays the process of selecting eligible studies. Initially, 1180 studies were identified through searches of the PubMed, Cochrane, and EMBASE databases. After removing duplicates, 722 studies remained. After reviewing titles and abstracts, 46 studies were selected for full-text review. Finally, 19 studies that met the inclusion criteria were included in this meta-analysis (4, 12–29).

### 3.2 Study characteristics and quality

This meta-analysis included 21 trials from 19 studies, involving 2097 patients with ARMCC. Among them, there were 15 observational studies (4, 12, 13, 15–19, 21, 22, 24, 25, 27–29), one expansion cohort study (23), and three RCTs (14, 20, 26). All studies included at least one group that received treatment with PD-1 inhibitors. Among the 21 trials, 10 involved the use of PD-1 inhibitor monotherapy (12, 13, 15, 19, 22–26, 29), five combined PD-1 inhibitors with chemotherapy (4, 12, 14, 18, 20), five combined PD-1 inhibitors with anti-angiogenic agents (16, 17, 27–29), and one combined PD-1 inhibitors with Cytotoxic T-lymphocyte antigen 4 (CTLA-4) inhibitors (21). Seven trials reported a correlation between the PD-L1 CPS and efficacy in patients with ARMCC (13, 20–22, 24, 25, 27). Eight trials reported a correlation between histological type and efficacy in patients (12, 21–23, 25, 26, 28, 29) (**Table 1**).

**Table 1:** Characteristics of studies included in this meta-analysis.

Trials name	year	Study type	Intervention drugs	Intervention types	Number of patients	Stage	PD-L1 CPS≥1%	PD-L1 CPS<1%	PD-L1 CPS unknown	Age, median (range)	Squamous cell carcinoma (%)	Follow-up(m), median (range)
Frenel	2017	NRCT Single arm	Pembrolizumab	PD-1 inhibitors monotherapy	24	Advanced or metastatic	24	0	0	42(26-62)	23 (95.8)	11(1.3-32.2)
Tamura	2019	NRCT Single arm	Nivolumab	PD-1 inhibitors monotherapy	20	Recurrent or advanced	15	5	0	50(32-68)	14 (70)	NR
Chung	2019	NRCT Single arm	Pembrolizumab	PD-1 inhibitors monotherapy	98	Advanced	82	15	1	46(24-75)	92 (93.9)	10.2(0.6-22.7)
Friedman	2020	NRCT Single arm	Atezolizumab + Bevacizumab	PD-1 inhibitors + Anti-angiogenic agent	11	Recurrent or metastatic or persistent	NR	NR	NR	48(31-55)	6(54.5)	NR
Rischin	2020	NRCT	Cemiplimab	PD-1 inhibitors monotherapy	10	Recurrent or metastatic	NR	NR	NR	55(31-76)	4 (40)	5.6(0.8-16.2)
O’Malley	2021	NRCT Single arm	Balstilimab	PD-1 inhibitors monotherapy	140	Recurrent or metastatic or persistent	85	38	17	53 (25–81)	85 (60.7)	14.6 (9.9–38.8)
Miller	2021	NRCT Single arm	Pembrolizumab	PD-1 inhibitors monotherapy	14	Recurrent	13	0	1	59 (22-77)	11 (78.5)	14.4 (3.3–39.0)
Huang	2021	NRCT Single arm	Camrelizumab + Apatinib	PD-1 inhibitors + Anti-angiogenic agent	32	Recurrent or metastatic or persistent	35	6	1	50 (33–63)	21 (65.6)	NR
Santin	2021	NRCT Single arm	Nivolumab	PD-1 inhibitors monotherapy	25	Recurrent or persistent	17	5	3	45 (20-79)	15(60)	32(2-41.5)
Colombo	2021	RCT	Pembrolizumab + Paclitaxel + Cisplatin or Carboplatin Versus Paclitaxel + Cisplatin or Carboplatin	PD-1 inhibitors + Chemotherapy Versus Chemotherapy alone	617	Recurrent or metastatic or persistent	273	35	0	51(25-82)	235 (76.3)	22(15.1-29.4)
O’Malley	2022	NRCT Single arm	Balstilimab + Zalifrelimab	PD-1 inhibitors + CTLA-4 inhibitor	125	Recurrent or/and metastatic	67	33	25	50 (24-76)	89 (71.2)	21 (11.8-32.1)
Xia	2022	NRCT Single arm	Camrelizumab + Famitinib	PD-1 inhibitors + Anti-angiogenic agent	33	Recurrent or metastatic	10	9	14	50 (43–55)	33(100)	13.6(10-23.6)
Ma	2022	NRCT Single arm	Sintilimab or Tislelizumab or Camrelizumab + Paclitaxel + Cisplatin	PD-1 inhibitors + Chemotherapy	85	FIGO IVB stage or recurrent or metastatic	30	55	0	52 (46-62)	68 (80)	23.4 (22.19–24.62)
Cheng A	2022	NRCT Single arm	Camrelizumab or Sintilimab	PD-1 inhibitors	24	Recurrent or metastatic	NR	NR	NR	52 (22-78)	UTE	18 (2-28)
Cheng B	2022	NRCT Single arm	Camrelizumab or Sintilimab + Paclitaxel + Cisplatin or Carboplatin	PD-1 inhibitors + Chemotherapy	26	Recurrent or metastatic	NR	NR	NR	52 (22-78)	UTE	18 (2-28)
Tewari	2022	RCT	Cemiplimab Versus Pemetrexed or Topotecan or Irinotecan or Gemcitabine or Vinorelbine	PD-1 inhibitors Versus Chemotherapy	608	Recurrent or metastatic	82	44	178	51 (22–81)	240 (78.9)	18.2 (6.0 - 38.2)
Xu	2022	NRCT Single arm	Sintilimab + Anlotinib	PD-1 inhibitors + Anti-angiogenic agent	42	Recurrent or metastatic	42	0	0	53(36-67)	35 (83.3)	10.9(0.03-19.2)
An	2023	NRCT Single arm	Serplulimab + Nab-Paclitaxel	PD-1 inhibitors + Chemotherapy	21	Recurrent or/and metastatic	21	0	0	50.8 (31–64)	20 (95.2)	14.6(0.2-21.7)
Nishio	2023	RCT	Pembrolizumab + Paclitaxel + Cisplatin or Carboplatin Versus Paclitaxel + Cisplatin or Carboplatin	PD-1 inhibitors + Chemotherapy Versus Chemotherapy alone	57	Recurrent or metastatic or persistent	30	5	0	54 (26–82)	27(77.1)	23.2 (16.4-27.8)
Zheng A	2023	NRCT Single arm	Tislelizumab + Bevacizumab or Apatinib	PD-1 inhibitors + Anti-angiogenic agent	44	Recurrent or metastatic	NR	NR	NR	54 (32-70)	UTE	11.3 (2.2-28.7)
Zheng B	2023	NRCT Single arm	Tislelizumab	PD-1 inhibitors monotherapy	41	Recurrent or metastatic	NR	NR	NR	54 (32-70)	UTE	11.3 (2.2-28.7)

RCT, randomized controlled trial; NRCT, not RCT; PD-1, programmed cell death protein-1; PD-L1, programmed cell death ligand-1; ICIs, Immune Checkpoint Inhibitors; CPS, combined positive score; ECOG, Eastern Cooperative Oncology Group; NR, not report; UTE, Unable to extract.

As shown in **Table 2**, all 15 observational studies scored greater than 14 points. **Supplementary Table 1** shows that all three RCTs were of high quality. **Supplementary Table 2** shows that the quality assessment of the expansion cohort study yielded a score of nine points. It is noteworthy that all 19 studies mentioned above met the inclusion criteria.

**Table 2:** The IHE AQ checklist for evaluating the quality of eligible observational studies.

trails name	year	①	②	③	④	⑤	⑥	⑦	⑧	⑨	⑩	⑪	⑫	⑬	⑭	⑮	⑯	⑰	⑱	⑲	⑳	scores
An	2023	yes	yes	yes	yes	yes	yes	yes	yes	yes	yes	unclear	yes	yes	yes	yes	yes	yes	yes	yes	yes	19
Cheng	2022	yes	no	no	no	yes	yes	yes	yes	yes	yes	unclear	yes	yes	yes	yes	yes	yes	yes	yes	yes	16
Chung	2019	yes	yes	yes	yes	yes	yes	yes	yes	yes	yes	unclear	yes	yes	yes	yes	yes	yes	yes	yes	yes	19
Frenel	2017	yes	yes	yes	yes	yes	yes	yes	yes	yes	yes	unclear	yes	yes	yes	yes	yes	yes	yes	yes	yes	19
Friedman	2020	yes	yes	yes	yes	yes	yes	yes	yes	yes	yes	unclear	yes	yes	yes	yes	yes	no	yes	yes	yes	18
Huang	2021	yes	yes	yes	yes	yes	yes	yes	yes	yes	yes	unclear	yes	yes	yes	yes	yes	partial	yes	yes	yes	18
Ma	2022	yes	no	yes	no	yes	yes	yes	yes	yes	yes	unclear	yes	yes	yes	yes	yes	partial	yes	yes	yes	16
Miller	2021	yes	yes	no	no	no	yes	yes	yes	yes	yes	unclear	yes	yes	yes	yes	yes	no	no	yes	yes	14
O’Malley	2021	yes	yes	yes	yes	yes	yes	yes	yes	yes	yes	unclear	yes	yes	yes	yes	yes	yes	yes	yes	yes	19
O’Malley	2022	yes	yes	yes	yes	yes	yes	yes	yes	yes	yes	unclear	yes	yes	yes	yes	yes	yes	yes	yes	yes	19
Santin	2020	yes	no	no	no	yes	yes	yes	yes	yes	yes	unclear	yes	yes	yes	yes	yes	yes	yes	yes	yes	16
Tamura	2019	yes	yes	yes	yes	yes	yes	yes	yes	yes	yes	unclear	yes	yes	yes	yes	yes	yes	yes	yes	yes	19
Xia	2022	yes	yes	yes	yes	yes	yes	yes	yes	yes	yes	unclear	yes	yes	yes	yes	yes	yes	yes	yes	yes	19
Xu	2022	yes	yes	yes	yes	yes	yes	yes	yes	yes	yes	unclear	yes	yes	yes	yes	yes	yes	yes	yes	yes	19
Zheng	2023	yes	no	no	no	yes	no	yes	yes	yes	yes	unclear	yes	yes	yes	yes	yes	yes	yes	yes	yes	15

①: Was the hypothesis/aim/objective of the study clearly stated?

②: Was the study conducted prospectively?

③: Were the cases collected in more than one centre?

④: Were patients recruited consecutively?

⑤: Were the characteristics of the patients included in the study described?

⑥: Were the eligibility criteria (i.e. inclusion and exclusion criteria) for entry into the study clearly stated?

⑦: Did patients enter the study at a similar point in the disease?

⑧: Was the intervention of interest clearly described?

⑨: Were additional interventions (co-interventions) clearly described?

⑩: Were relevant outcome measures established a priori?

⑪: Were outcome assessors blinded to the intervention that patients received?

⑫: Were the relevant outcomes measured using appropriate objective/subjective methods?

⑬: Were the relevant outcome measures made before and after the intervention?

⑭: Were the statistical tests used to assess the relevant outcomes appropriate?

⑮: Was follow-up long enough for important events and outcomes to occur?

⑯: Were losses to follow-up reported?

⑰: Did the study provided estimates of random variability in the data analysis of relevant outcomes?

⑱: Were the adverse events reported?

⑲: Were the conclusions of the study supported by results?

⑳: Were both competing interests and sources of support for the study reported?

The criteria for quality rating scores are as follows: 1 point for a ‘yes’ answer, 0 for an ‘unclear’, ‘partial’ or ‘no’ answer.

The quality of included studies was evaluated based on 20 items from the Delphi checklist. If the literature≥14 items of the Delphi checklist, it was considered to meet acceptable quality criteria.

IHE QA, Institute of Health Economics Quality Appraisal.

### 3.3 Efficacy

#### 3.3.1 ORR in ARMCC patients

**Figure 2:**
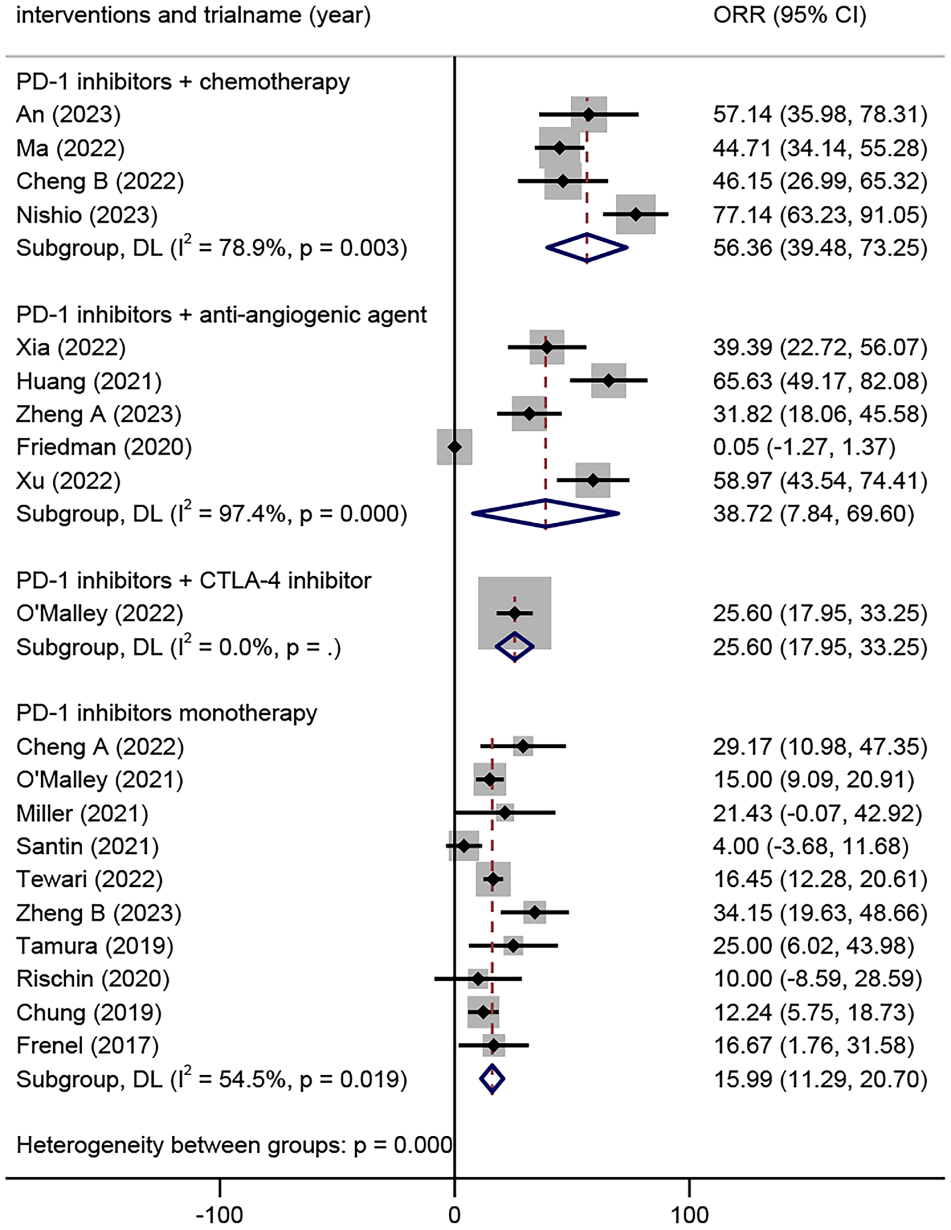
Overall objective response rates of different combinations of PD-1 inhibitors in the treatment of advanced, recurrent, or metastatic cervical cancer. PD-1, Programmed Cell Death Protein 1.

Twenty trials comprising 2040 patients were eligible for the ORR. The analyses based on intervention types indicated that the ORR of the combination of PD-1 inhibitors with chemotherapy was 56.36% (95% CI 39.48% to 73.25%), the combination of PD-1 inhibitors with anti-angiogenic agents was 38.72% (95% CI 7.84% to 69.60%), the combination of PD-1 inhibitors with CTLA-4 inhibitors was 25.60 (17.95, 33.25), and PD-1 inhibitor monotherapy was 15.99% (95% CI 11.29% to 20.70%) (**Figure 2**).

#### 3.3.2 Comparison of the ORR among different histological types of ARMCC patients

**Figure 3:**
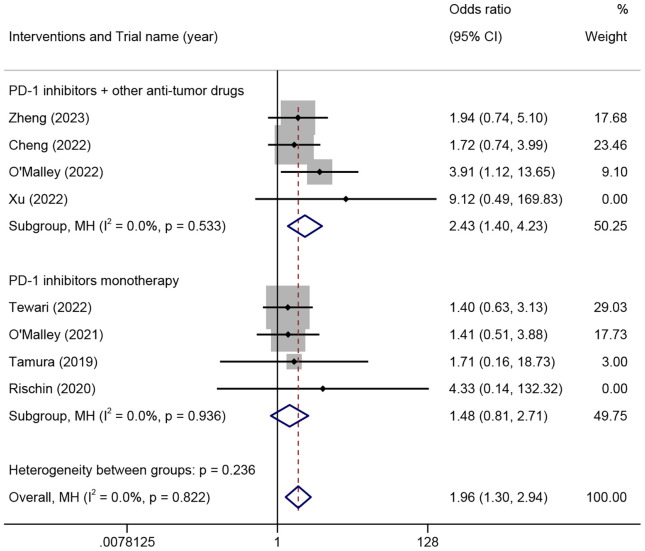
The odds ratio of objective response rates in squamous cell carcinoma and non-squamous cell carcinoma patients treated with different combinations of PD-1 inhibitors for advanced, recurrent, or metastatic cervical cancer. PD-1, programmed cell death protein 1.

The ORR among patients with different histological types of ARMCC was analyzed in eight trials comprising 1080 patients. The results demonstrated that there was no significant difference in the ORR between the SSC and non-SSC groups in patients who received PD-1 inhibitor monotherapy (OR=1.48, 95% CI 0.81-2.71, P=0.203, I²=0%). However, the SSC group exhibited a significantly higher ORR compared to the non-SSC group in patients who received PD-1 inhibitors combined with other anti-tumor drugs (OR=2.43, 95% CI 1.40-4.23, P=0.002, I²=0%) (**Figure 3**).

#### 3.3.3 Comparison of the ORR among different PD-L1 CPS of ARMCC patients

**Figure 4:**
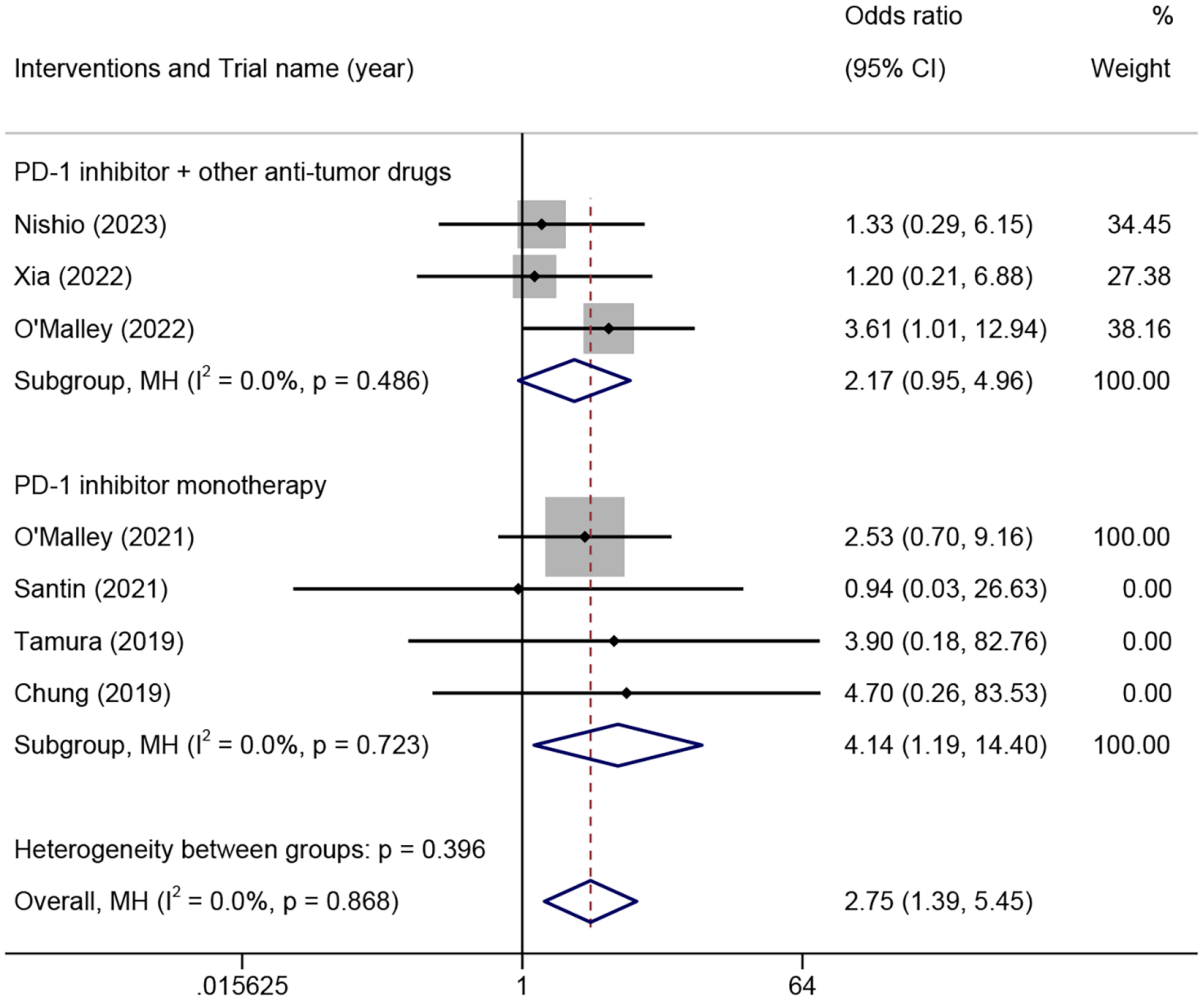
The odds ratio of objective response rates in PD-L1 combined positive score ≥1 and <1 patients treated with different combinations of PD-1 inhibitors for advanced, recurrent, or metastatic cervical cancer. PD-L1, programmed death-ligand 1; PD-1, programmed cell death protein 1.

The ORR among different PD-L1 CPS of patients was analyzed in seven trials involving 498 patients. The results demonstrated that the PD-L1 CPS ≥1 group exhibited a significantly higher ORR compared to the PD-L1 CPS <1 group in patients who received PD-1 inhibitor monotherapy (OR=4.14, 95% CI 1.19-14.40, P=0.02, I²=0%) (**Figure 4**). Additionally, the PD-L1 CPS ≥1 group exhibited a higher ORR compared to the PD-L1 CPS <1 group, but there was no statistical difference in patients who received PD-1 combined with other anti-tumor drugs (OR=2.17, 95% CI 0.95-4.96, p=0.067, I²=0%) (**Figure 4**).

#### 3.3.4 1-year OS rate and 1-year PFS rate in ARMCC patients

**Figure 5:**
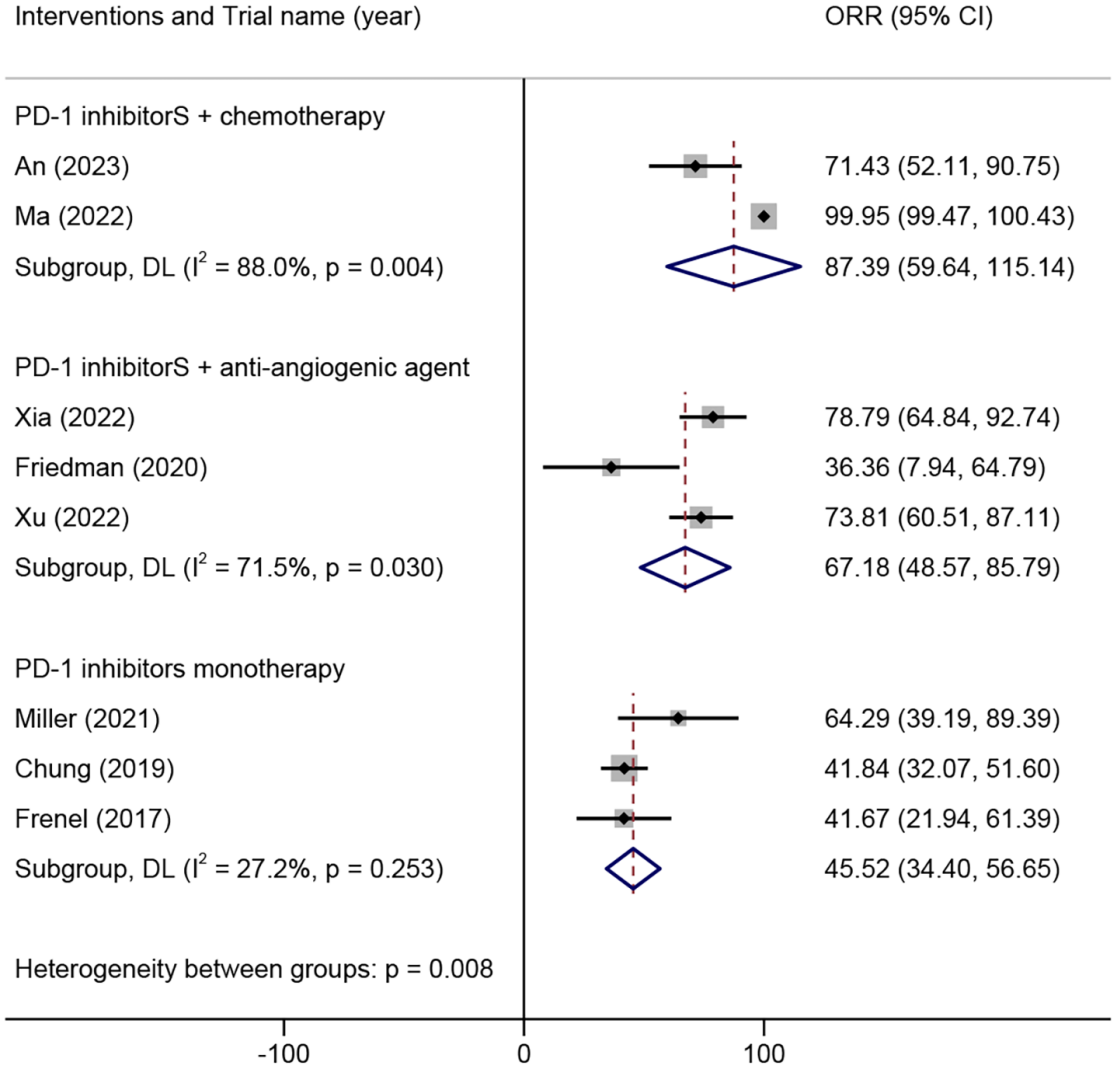
1-year overall survival rate of different combinations of PD-1 inhibitors in the treatment of advanced, recurrent, or metastatic cervical cancer. PD-1, programmed cell death protein 1.

Eight trials including 328 patients were eligible for inclusion based on the 1-year OS rate. The analysis based on different intervention types indicated that the 1-year OS rate in ARMCC patients who received PD-1 inhibitors combined with chemotherapy was 87.39% (95% CI 59.64%-115.14%), that in ARMCC patients who received PD-1 inhibitors combined with anti-angiogenic agents was 67.18% (95% CI 48.57%-85.79%), and that in ARMCC patients who received PD-1 inhibitor monotherapy was 50.0% (95% CI 39.0%-61.0%) (**Figure 5**).

Five trials including 177 patients were eligible for inclusion based on the 1-year PFS rate. The analysis based on different intervention types indicated that the 1-year PFS rate in patients with ARMCC who received PD-1 inhibitors combined with chemotherapy was 58.85% (95% CI 42.96%-74.75%), that in patients with ARMCC who received PD-1 inhibitors combined with anti-angiogenic agents was 48.48% (95% CI 31.43%-65.54%), and that in patients with ARMCC who received PD-1 inhibitor monotherapy was 17.61% (95% CI -12.97%-48.18%) (**Supplementary Figure 1**).

#### 3.3.5 The HRs for OS and PFS in RCTs

Two RCTs including 674 patients were eligible for OS and PFS analyses. The OS was significantly higher in ARMCC patients who received PD-1 inhibitors with chemotherapy compared to those who received chemotherapy alone (HRs=0.65, 95% CI 0.53-0.81, p=0.000, I²=10%) (**Supplementary Figure 2**). Similarly, the PFS in ARMCC patients who received PD-1 inhibitors with chemotherapy was significantly higher compared to those who received chemotherapy alone (HRs=0.63, 95% CI 0.52-0.77, p=0.000, I²=0%) (**Supplementary Figure 3**).

### 3.4 Safety

#### 3.4.1 Overall incidence of AEs

This review included 17 studies reporting AEs, with 85 different types of AEs included in 4049 cases.

**Figure 6:**
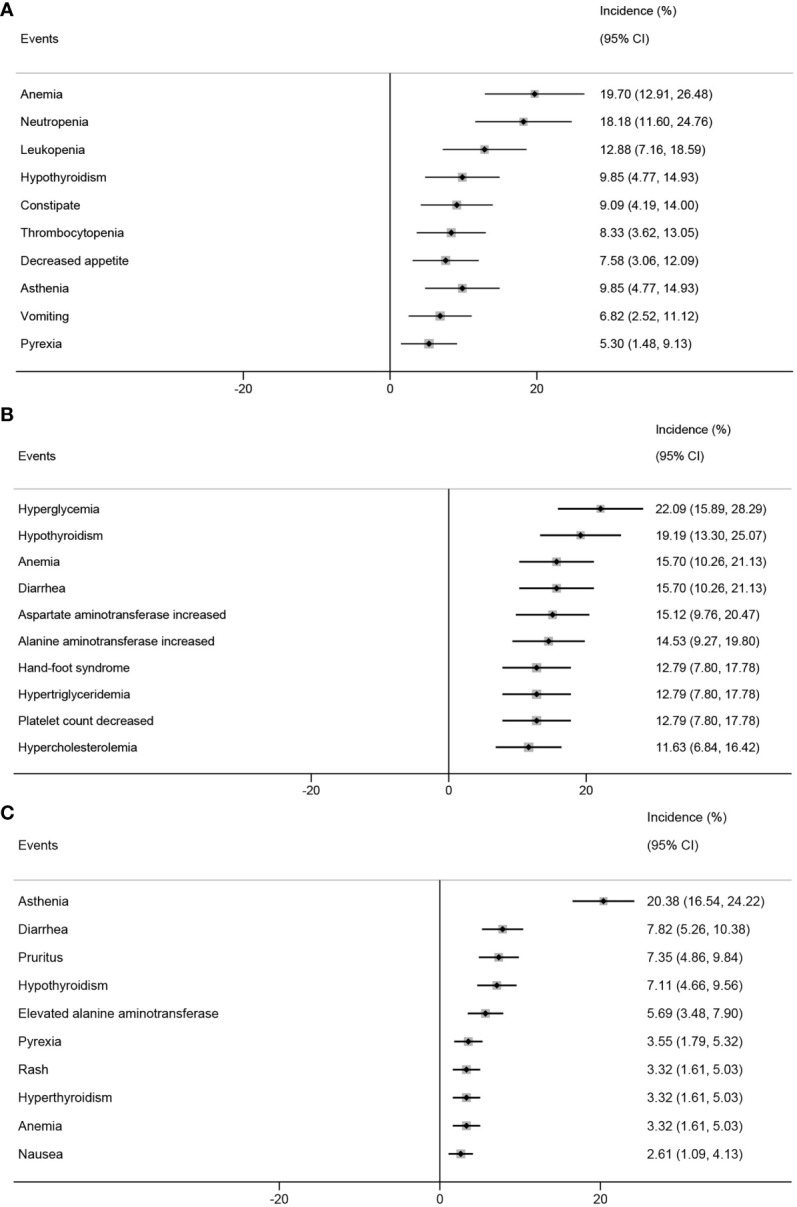
Top 10 incidence of adverse events in patients with advanced, recurrent, or metastatic cervical cancer treated with different combinations of PD-1 inhibitors. PD-1 inhibitors combined with chemotherapy **(A)**, with anti-angiogenic agent **(B)**, monotherapy **(C)**. PD-1, programmed cell death protein 1.

In the analysis based on the intervention type, the top five AEs in patients who received PD-1 inhibitors combined with chemotherapy were anemia (19.70%; 95% CI, 12.91%-26.48%), neutropenia (18.18%; 95% CI, 11.60%-24.76%), leukopenia (12.88%; 95% CI, 7.16%-18.59%), hypothyroidism (9.85%; 95% CI, 4.77%-14.93%), and constipation (9.09%; 95% CI, 4.19%-14.00%) (**Figure 6A**). The top five AEs in patients who received PD-1 inhibitors combined with anti-angiogenic agents were hyperglycemia (22.09%; 95% CI, 15.89%-28.29%), hypothyroidism (19.19%; 95% CI, 13.30%-25.07%), anemia (15.70%; 95% CI, 10.26%-21.13%), diarrhea (15.70%; 95% CI, 10.26%-21.13%), and elevated aspartate aminotransferase levels (15.12%; 95% CI, 9.76%-20.47%) (**Figure 6B**). The top five AEs in patients who received PD-1 inhibitor monotherapy were asthenia (20.38%; 95% CI, 16.54%-24.22%), diarrhea (7.82%; 95% CI, 5.26%-10.38%), pruritus (7.35%; 95% CI, 4.86%-9.84%), hypothyroidism (7.11%; 95% CI, 4.66%-9.56%), and elevated alanine transaminase (ALT) levels (5.69%; 95% CI, 3.48%-7.90%) (**Figure 6C**).

#### 3.4.2 Incidence of immune-related AEs

PD-1 inhibitors block the immune checkpoint pathway, reactivate cellular immunity, and cause autoimmune-mediated AEs. This study included 10 studies reporting 33 different types of ir-AEs involving 314 cases.

**Figure 7:**
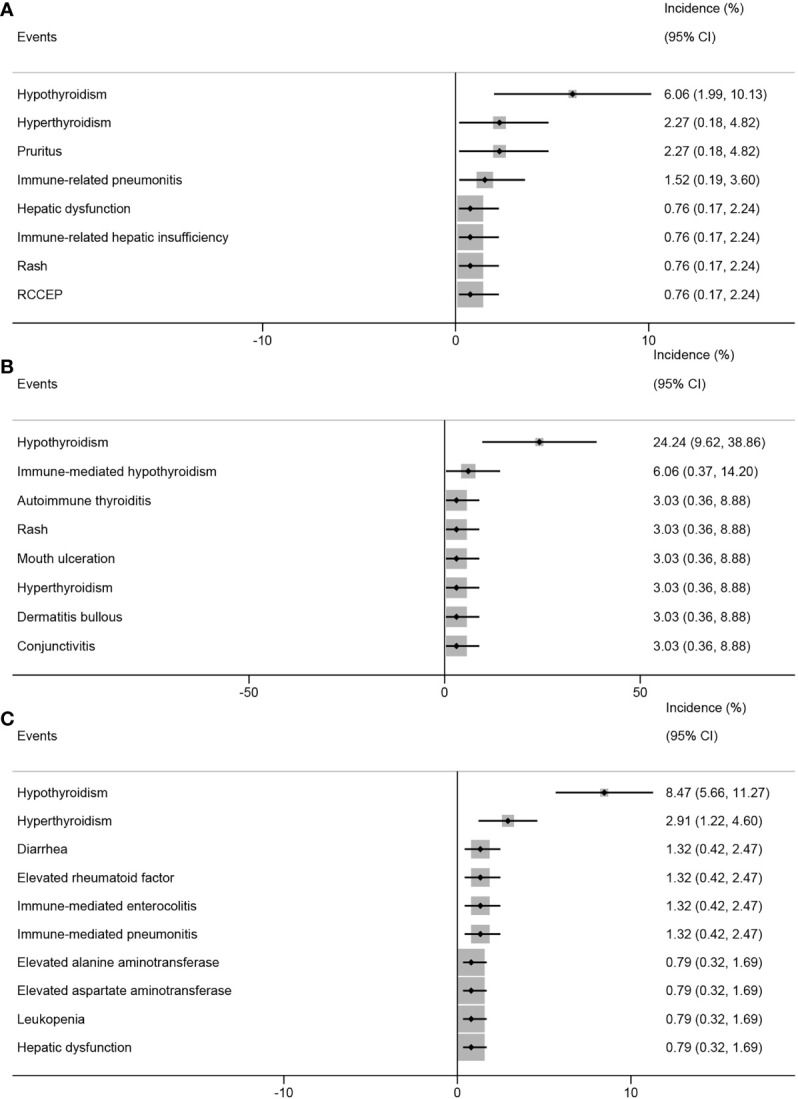
Top 8 incidence of immune-related adverse events in patients with advanced, recurrent, or metastatic cervical cancer treated with different combinations of PD-1 inhibitors. PD-1 inhibitors combined with chemotherapy **(A)**, with anti-angiogenic agent **(B)**, monotherapy **(C)**. PD-1, programmed cell death protein 1.

In the analysis based on the intervention type, the top three ir-AEs in patients who received PD-1 inhibitors combined with chemotherapy were hypothyroidism (6.06%; 95% CI, 1.99%-10.13%), hyperthyroidism (2.27%; 95% CI, 0.18%-4.82%), and pruritus (2.27%; 95% CI, 0.18%-4.82%) (**Figure 7A**). The top three ir-AEs in patients who received PD-1 inhibitors combined with anti-angiogenic agents were hypothyroidism (24.24%; 95% CI, 9.62%-38.86%), immune-mediated hypothyroidism (6.06%; 95% CI, 0.37%-14.20%), and autoimmune thyroiditis (3.03%; 95% CI, 0.36%-8.88%) (**Figure 7B**). The top three ir-AEs in patients who received PD-1 inhibitor monotherapy were hypothyroidism (8.47%; 95% CI, 5.66%-11.27%), hyperthyroidism (2.91%; 95% CI, 1.22%-4.60%), and diarrhea (1.32%; 95% CI, 0.42%-2.47%) (**Figure 7C**).

#### 3.4.3 Overall incidence of AEs in RCTs

**Figure 8:**
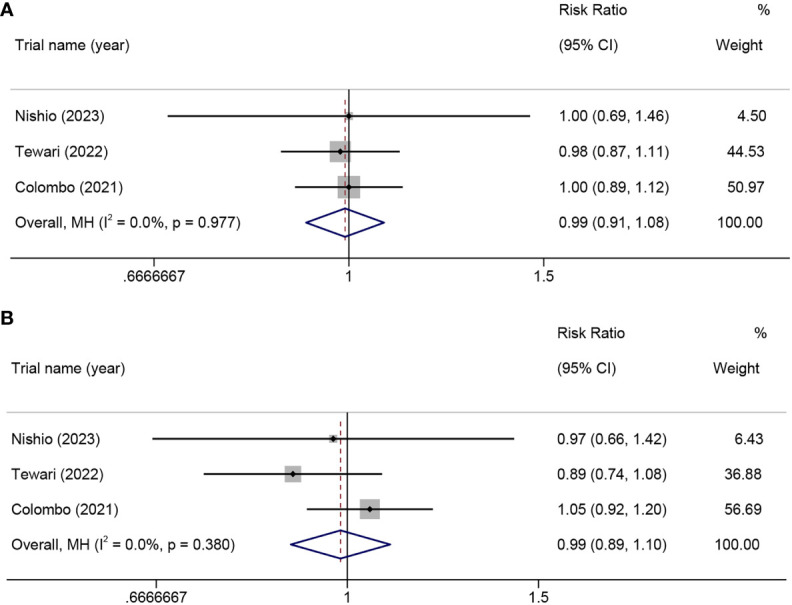
In a randomized controlled trial, the risk ratio of adverse events in patients with advanced, recurrent, or metastatic cervical cancer treated with PD-1 inhibitors combined with chemotherapy versus chemotherapy alone. all-grade adverse events **(A)**, grade ≥3 adverse events **(B)**. PD-1, programmed cell death protein 1.

In this meta-analysis including three RCTs, the results indicated that PD-1 inhibitor monotherapy or PD-1 inhibitors combined with chemotherapy did not significantly increase the incidence of all grades of AEs (RR=0.99, 95% CI 0.91-1.08, p=0.788, I²=0.0%) (**Figure 8A**) or the incidence of serious AEs (grade≥3) (RR=0.99, 95% CI 0.89-1.10, p=0.788, I²=0.0%) when compared to chemotherapy alone (**Figure 8B**).

### 3.5 Publication bias

No significant publication bias was detected in the funnel plot, and the p-values of Egger’s test for the pooled ORR in different histological types or PD-L1 CPS of patients with ARMCC were not significant (p=0.083 and p=0.709, respectively) (**Supplementary Figures 4**, **5**).

## 4 Discussion

The binding of PD-L1, which is expressed on tumor cells or within the tumor microenvironment, to PD-1 on T cells leads to the suppression of T cell function, allowing cancer cells to evade immune surveillance and promote tumor development. Conversely, PD-1 inhibitors can reverse T cell dysfunction or apoptosis and maintain peripheral immune system tolerance, thereby exerting anti-tumor effects. This treatment strategy has shown significant success in the treatment of various malignant tumors and is transforming cancer therapy (5–7). As a significant category, PD-1 inhibitors have been approved for the treatment of various malignancies, including non-small cell lung cancer, malignant melanoma, and CC (30–33).

However, the therapeutic efficacy of PD-1 inhibitors for the treatment of CC, particularly ARMCC, remains controversial (20, 26). The key controversial issues include the following:1) Can the combination of PD-1 inhibitors with other treatment modalities significantly improve the therapeutic efficacy? For example, a combination of chemotherapy, anti-angiogenic agents, or other immunotherapies can effectively control tumor growth and spread. 2) Does the use of PD-1 inhibitors lead to severe adverse events? These adverse events can significantly affect the quality of life of patients with CC. Therefore, we conducted this study to address these issue. To the best of our knowledge, this study represents the first comprehensive analysis on the effectiveness and safety of PD-1 inhibitors combined with other anti-tumor drugs for the treatment of patients with ARMCC.

We found that, among the several treatment strategies for PD-1 inhibitor therapy in ARMCC, the combination of PD-1 inhibitors with chemotherapy exhibited the highest ORR. The combination of PD-1 inhibitors with anti-angiogenic agents exhibited the second highest ORR, followed by the combination of PD-1 inhibitors and CTLA-4 inhibitors. Finally, PD-1 inhibitor monotherapy resulted in the lowest ORR. In terms of the 1-year OS and 1-year PFS, various treatment strategies involving PD-1 inhibitor therapy in ARMCC have yielded similar results.

Based on the above results, it can be inferred that the treatment outcomes in ARMCC tend to favor the combination of PD-1 inhibitors with other anti-tumor drugs, such as chemotherapy, anti-angiogenic agents, or CTLA-4 inhibitors, rather than PD-1 inhibitor monotherapy. Evidence supports the suggestion that chemotherapy drugs and anti-angiogenic agents are able to disrupt tumor cells and release immunostimulatory tumor antigens, thereby enhancing immunogenicity (34). Our findings also confirmed that combination therapy enhanced the anti-tumor effects of PD-1 inhibitors.

According to a stratified analysis based on histological types and PD-L1 positive expression in ARMCC, the results revealed that in PD-1 monotherapy, the SCC and non-SCC groups did not exhibit a significant difference in terms of ORR. However, the group with a CPS≥1 exhibited a significantly higher ORR compared to the CPS<1 group. Additionally, when PD-1 inhibitors were used in combination with other anti-tumor drugs, the SCC group exhibited a significantly higher ORR than the non-SCC group. Moreover, the CPS≥1 group exhibited a higher ORR than the CPS<1 group, although the difference was not statistically significant. These findings suggest that SCC patients may benefit more from combined treatment with PD-1 inhibitors and other anti-tumor drugs, and patients with positive PD-L1 expression exhibit a better response to immunotherapy, which suggests that PD-L1 may be a potential biomarker for predicting clinical outcomes in patients with cervical cancer. Although a previous study indicated that PD-L1 expression levels did not enhance the OS and PFS of patients with ARMCC treated with PD-1 inhibitors (31), it is important to note that assessing the impact of drugs on OS and PFS can be confounded by the complex nature of the causes of death in patients with ARMCC, potentially introducing bias into the results. In contrast, ORR reflects the proportion of tumors that experience a rapid reduction or disappearance in volume within a short period of time, which provides a better indication of the therapeutic effect of drugs on tumors.

To enhance the credibility of our research findings, we conducted a meta-analysis of RCTs. The findings demonstrated that, compared to patients undergoing chemotherapy alone, the combination of PD-1 inhibitors with chemotherapy can significantly improve OS and PFS in patients with ARMCC. These results further support the superior efficacy of PD-1 inhibitors combined with chemotherapy compared to chemotherapy alone, and that more patients with ARMCC could benefit from them.

We also conducted a safety analysis and found that, on average, each patient with ARMCC who received PD-1 inhibitor treatment experienced approximately two adverse events. It is crucial to communicate these statistical data to patients before initiating PD-1 inhibitor therapy. The top five AEs in patients who received PD-1 inhibitor monotherapy were asthenia, diarrhea, hypothyroidism, pruritus, and elevated alanine aminotransferase levels. Similarly, patients who received PD-1 inhibitors in combination with chemotherapy experienced anemia, neutropenia, leukopenia, hypothyroidism, and constipation. Furthermore, patients who received PD-1 inhibitors combined with anti-angiogenic agents experienced hyperglycemia, hypothyroidism, anemia, diarrhea, and elevated aspartate aminotransferase levels. The top three ir-AEs in patients who received PD-1 inhibitor monotherapy were hypothyroidism, hyperthyroidism, and diarrhea. Similarly, patients who received PD-1 inhibitors in combination with chemotherapy experienced hypothyroidism, hyperthyroidism, and pruritus. Lastly, patients who received PD-1 inhibitors in combination with anti-angiogenic agents experienced hypothyroidism, immune-mediated hypothyroidism, and autoimmune thyroiditis.

We included three RCTs in the safety analysis to assess whether the use of PD-1 inhibitors would increase the incidence of AEs. The results showed that PD-1 inhibitor monotherapy or PD-1 inhibitors in combination with chemotherapy did not increase the overall or severe AEs rates compared to chemotherapy alone. These findings are similar to a previous large-scale meta-analysis, which indicates the reliability of our analysis, despite our focus on only analyzing the AEs reported in the included literature (35).

This study has some limitations. First, most of the included studies were single-arm trials, which introduced a certain risk of bias and confounding factors owing to the lack of a control group. Even though we attempted to mitigate these risks through subgroup and stratified analyses, significant heterogeneity remained in some of the results. Second, in some of the included studies, data such as OS and PFS could not be utilized as the patients did not reach the median survival time, resulting in a less comprehensive survival analysis. Third, only one study on the use of PD-L1 inhibitors in combination with CTLA-4 inhibitors was included. Therefore, it is crucial to continue to monitor research related to PD-L1 inhibitors combined with CTLA-4 inhibitors to obtain more data that can be used to validate our results. Fourth, variations in PD-1 inhibitor use across studies in terms of therapy lines, combination regimens, treatment durations, and dosages may have increased outcome heterogeneity. Despite the significant heterogeneity, this study still holds value and significance.

## 5 Conclusion

Our study revealed that PD-1 inhibitors demonstrate outstanding efficacy in the treatment of patients with ARMCC. Patients with SCC may benefit more from treatments including PD-1 inhibitors in combination with other anti-tumor drugs. Additionally, PD-L1 may be a potential biomarker for predicting clinical outcomes in patients with cervical cancer. Importantly, the use of PD-1 inhibitor monotherapy or PD-1 inhibitors in combination with chemotherapy did not lead to an increased incidence of AEs compared with chemotherapy alone, indicting safety during. Furthermore, identifying more subgroups of cervical cancer that benefit from PD-1 inhibitors is a direction worth researching.

## Data availability statement

The original contributions presented in the study are included in the article/**Supplementary Material**. Further inquiries can be directed to the corresponding author.

## Author contributions

YW: Writing – original draft, Writing – review & editing. JW: Writing – original draft. JD: Writing – original draft. XT: Writing – original draft. JX: Writing – original draft, Writing – review & editing.
