# Supplementary material for: Clinical benefit analysis of PD-1 inhibitors in patients with advanced, recurrent or metastatic cervical cancer: a meta-analysis and systematic review
Source: Front Immunol. 2024 Jan 24;15:1305810. doi: 10.3389/fimmu.2024.1305810 (PMC10847356; doi:10.3389/fimmu.2024.1305810)
Supplement: Supplementary file 1 [file DataSheet_1.zip › Supplementary Figure + Table.doc]

# 1 Supplementary Figures and Tables

## 1.1 Supplementary Figures

**Supplementary Figure 1.** 1-year progression-free survival rate of different combinations of PD-1 inhibitors in the treatment of advanced, recurrent, or metastatic cervical cancer. PD-1, programmed cell death protein 1.

**Supplementary Figure 2.** The hazard ratio of overall survival in patients with advanced, recurrent, or metastatic cervical cancer treated with PD-1 inhibitors with chemotherapy compared to those who received chemotherapy alone. PD-1, programmed cell death protein 1.

**Supplementary Figure 3.** The hazard ratio of progression-free survival in patients with advanced, recurrent, or metastatic cervical cancer treated with PD-1 inhibitors with chemotherapy compared to those who received chemotherapy alone. PD-1, programmed cell death protein 1.

**Supplementary Figure 4.** The funnel plot in squamous cell carcinoma and non-squamous cell carcinoma patients treated with different combinations of PD-1 inhibitors for advanced, recurrent, or metastatic cervical cancer. PD-1, programmed cell death protein 1.

**Supplementary Figure 5.** The funnel plot in PD-L1 combined positive score ≥1 and <1 patients treated with different combinations of PD-1 inhibitors for advanced, recurrent, or metastatic cervical cancer. PD-L1, programmed death-ligand 1; PD-1, programmed cell death protein 1.

## 1.2 Supplementary Tables

| **Supplementary Table 1.** Cochrane Risk of Bias Assessment Tool for assessing the quality of randomized controlled trials in meta-analyses. | | | | | | | | | |
| --- | --- | --- | --- | --- | --- | --- | --- | --- | --- |
| Trails name | Year | Random sequence generation (selection bias) | Allocation concealment (selection bias) | Blinding of participants and personnel (performance bias) | Blinding of outcome assessment (detection bias) | Incomplete outcome data (attrition bias) | Selective reporting (reporting bias) | Other bias | Total quality scores |
| Colombo | 2021 | Low | Low | Low | Low | Low | Low | Low | High |
| Tewari | 2022 | Low | Low | Low | Low | Low | Low | Low | High |
| Nishio | 2023 | Low | Low | Low | Low | Low | Low | Low | High |

| **Supplementary Table 2.** Newcastle-Ottawa Scale for assessing the quality of nonrandomized studies in meta-analyses. | | | | | | | | | | |
| --- | --- | --- | --- | --- | --- | --- | --- | --- | --- | --- |
| Trails name | Year | Representativeness of the exposed cohort | Selection of the unexposed cohort | Ascertainment of exposure | Outcome of interest not present at start of study | Control for important factor or additional factor | Outcome assessment | Follow up long enough for outcomes to occur | Adequacy of follow up of cohorts | Total quality scores |
| Rischin | 2020 | +* | + | + | + | + | + | + | + | 8 |
| * If there is a positive symbol that means score one point  ** A negative symbol means no point | | | | | | | | | | |
